# Cooling Performance and Thermal Radiation Model of Asphalt Mixture with Modified Infrared Powder

**DOI:** 10.3390/ma14020245

**Published:** 2021-01-06

**Authors:** Lei Gao, Yanping Liu, Jianguang Xie, Zhaoxu Yang

**Affiliations:** 1 Department of Civil Engineering, Nanjing University of Aeronautics and Astronautics, Nanjing 210016, China; glzjy@nuaa.edu.cn (L.G.); spongeboblyp@nuaa.edu.cn (Y.L.); 2 Henan Provincial Department of Transportation, Zhengzhou 450016, China; aotuskyer@163.com

**Keywords:** modified infrared powder, cooling performance, asphalt mixture, pavement equilibrium temperature, infrared radiation transmissivity, shortwave absorptivity

## Abstract

This research studied a new material named modified infrared powder (MIRP) for decreasing the high temperature of asphalt pavements which can help alleviate the urban heat island effect to some extent. Based on the physical apparent density tests of materials and infrared thermal radiation test, the cooling performance of MIRP was obtained. X-ray diffraction analysis and scanning electron microscopy test (SEM) were conducted to analyze the chemical composition and the microstructure of MIRP, respectively. According to the radiant heat transfer theory, a thermal radiation model of the pavement equilibrium temperature was established by microscopic and chemical analysis to study the influence of thermal radiation asphalt mixture and reveal its cooling performance. The results show that the main components of MIRP are metal oxides and nonmetallic oxides which improve its infrared emissivity. Compared with limestone mineral powder asphalt mortar, the asphalt mortar with MIRP had a more compact structure and uniform distribution, and enhanced the overall structural performance of the mixture. The thermal radiation model reveals that the pavement equilibrium temperature combined with the MIRP in asphalt mixture decreases with the increase of the longwave emissivity, and it diminishes with the decrease of the shortwave absorptivity.

## 1. Introduction

The Urban Heat Island (UHI) phenomenon explains the undesirable situation whereby the urban areas’ temperature is higher than the surrounding suburban and rural areas, which has recently been of great concern [1,2]. The UHI effect on the urban environment is related to the high temperature destruction of urban asphalt pavement which has several adverse effects on urban construction [3]. Statistics and scientific studies show that summer temperatures in cities are 6 °C higher than in suburbs [4,5]. Together with anthropogenic activities, the phenomenon of global climate changes is also a key contributor to the observed rise in urban temperatures [6]. Alongside implementation of green roofs and vegetation covers, cool pavements have been recommended for urban temperature reduction [7]. Scholars across the world are currently working on ways to slow down the urban UHI effect and have proposed lots of techniques, including the use of highly reflective cooling pavement materials [8], such as adopting materials to amortize and dissipate solar energy [9], developing appropriate heat sinks to dissipate excess environmental energy with specific cooling materials [10], and these strategies minimize the high rate of heat absorption by highway pavements.

Overall, previous research findings have revealed that studying pavement materials and reducing the equilibrium temperature of the pavement are essential to mitigating urban thermal balance and solving the UHI effect [11]. Asphalt mixture is widely used in the pavement for its good road performance, low maintenance costs and noise reduction [12,13]. Besides, asphalt has a high heat absorption capacity, resulting in high temperature of asphalt pavement, which can reach 48–67 °C in summer [14]. Most innovative asphalt pavement designs that have been implemented across the world primarily aimed at reducing pavement surface temperature [15], which is mainly achieved by using pavement surfaces with high albedo to improve its performance reflectivity [16], utilizing water retentive pavement surfacing materials to increase evaporative cooling, converting the pavement heat to sustainable electrical energy and adding some new materials in the asphalt mixtures [17,18]. Earlier study has demonstrated that the potential for reducing pavement temperatures is through the use of high reflective materials, and low-thermal-radiation asphalt pavement can significantly lower the equilibrium temperature and improve the heat-storage capacity [13]. Meanwhile, road performance tests proved that infrared powder can enhance the performance of asphalt mixture and strengthen the high-temperature stability of the pavement [19].

The infrared powder material selected for this study is called modified infrared powder (MIRP), which is based on industrial waste and mineral raw materials, such as Vanadium-Titanium slag (VT-slag), Silico-Ferrite-slag (SF-slag), Ferrotitanium-slag ( TF-slag) as the basic raw material of infrared radiation base material, with high performance oxide material as the external admixture component, and the synthetic infrared powder material with high emissivity crystalline phase is formed by high temperature sintering process. MIRP is a synthetic infrared ceramic powder with black appearance and particle size of 700–800 mesh, which has the advantages of low price and high infrared emissivity. Adding an appropriate amount of MIRP to the asphalt mixture, the heat accumulation of the asphalt pavement can be reduced so that the pavement can convert the radiant energy into the atmospheric infrared window band and increase its atmospheric window band emissivity to reflect the pavement heat absorption to the cosmic space as much as possible, lowering the heat input to the atmosphere from the pavement and the pavement temperature. In this paper, the authors aim at revealing the cooling performance of modified infrared powder (MIRP) and establishing the thermal radiation model of the asphalt pavement equilibrium temperature. The cooling performance of MIRP was obtained by the asphalt slurry cooling performance test and infrared thermal radiation test. The chemical composition and the microstructure of MIRP were conducted by X-ray diffraction analysis and scanning electron microscopy test (SEM), respectively. A thermal radiation model of the pavement equilibrium temperature was established by microscopic and chemical analysis to study the influence of thermal radiation asphalt mixture and reveal its cooling performance.

## 2. Materials and Experimental Tests

### 2.1. Physical Properties Tests of Materials

The low heat accumulation asphalt mixture is a self-heating and cooling asphalt mixture prepared by mixing MIRP in the common asphalt mixture to replace part of the limestone mineral powder as the mixture filler. Due to the difference in physical properties between MIRP and limestone mineral powder, the size of the substitution rate will inevitably cause a change in the gradation, thus making the performance of asphalt mixture changed. Therefore, tests included the mineral apparent density test, the infrared emissivity test and asphalt slurry cooling performance test are conducted to study the physical properties of MIRP in Stone Matrix Asphalt(SMA) mixtures.

#### 2.1.1. Mineral Apparent Density Test

The surface dry relative density of coarse aggregates in SMA mixtures are measured by the Surface-dry Condition Method using an immersion balance, and the test data are shown in Table 1.

And the apparent relative density of fine aggregates in SMA mixtures are obtained by the measuring cylinder method, and the test data are recorded in Table 2.

#### 2.1.2. Infrared Emissivity Test

Infrared emissivity is the most essential property of infrared radiation material, which evaluates the infrared radiation performance of the material. The infrared emissivity test on the MIRP is using the IRE-1 infrared radiation measuring instrument developed by the Shanghai Institute of Technology Physics, Chinese Academy of Sciences, and the test temperature is room temperature.

The IRE-1 infrared radiation measuring instrument is mainly composed of six parts: optical system, infrared filter set, heat pipe furnace and precise temperature automatic control system, infrared detection and signal processing system, computer automatic control and data display system and optical machinery. The working principle of the IRE-1 infrared radiation measuring instrument is based on the radiant energy comparison method, that is, to compare the sample to be tested with a reference body with a known temperature and a known emissivity. The ratio of the radiant flux density of the black body cavity is the specific emissivity of the sample surface. According to the basic calculation formula of infrared radiation measuring instrument as follows:

(1) $$\varepsilon_{1}\left(T\right)=\frac{V}{V_{b}}\times \varepsilon_{b}$$

Since the normal emissivity of the standard sample is known, the radiation signal from the standard sample or the sample to be tested is modulated by the optical chopper, then collected and projected onto the pyroelectric detector by the ellipsoidal coaxial reflection optical system. The detector converts this alternating signal into an electrical signal, which is amplified by phase-lock and enters the computer for processing, thereby measuring the response V and $V_{b}$ of the instrument when the sample to be tested and the standard sample enter the optical path, and the synthetic infrared powder is made into ϕ20 × 1 mm of the standard sample, the infrared emissivity and wavelength are measured by IRE-1 infrared radiation measuring instrument, which can be seen in Table 3.

In Table 3, F_1_ is the full wavelength integrated emissivity; F_2_ is the (8–14) μm integrated emissivity; F_3_, F_4_, F_5_, F_6_, F_7_ are respectively the 8.45 μm, 9.50 μm, 10.60 μm, 12.00 μm, 13.50 μm Center wavelength, narrow band emission with bandwidth of 1 μm; F_8_—(14–25) μm integrated emissivity. Furthermore, from the data in Table 3, it can be concluded that the emissivity of MIRP in the 8–14 μm band reaches 0.94, while the emissivity at full wavelength is only 0.92. It reveals that the emissivity outside the atmospheric window is lower than 0.92, therefore the MIRP can be regarded as a certain degree of wavelength selective emission material.

#### 2.1.3. Asphalt Slurry Cooling Performance Test

In this study, industrial waste residue was selected as the basic material of MIRP, and high-performance oxide materials were used as the external components to form high-emissivity crystalline phases. In order to analyze the infrared radiation performance of MIRP wrapped in the asphalt film and study its cooling characteristics, a certain amount of MIRP and limestone mineral powder were mixed into the asphalt to prepare asphalt mortar. Then, to investigate the infrared radiation performance of MIRP under asphalt film encapsulation, a certain amount of MIRP and limestone mineral powder were mixed into asphalt to prepare asphalt mortar. Considering that the powder-to-glue ratio of SMA mixture generally reaches 1.8–2.0 level, and the need to maximize the proportion of filler in the mixture to achieve the best cooling effect, asphalt slurry powder-to-glue ratio of 2 was selected, that is, filler (MIRP + limestone mineral powder):asphalt = 2:1. The asphalt slurry specimen proportion is shown in Table 4, and the sample amounts are the proportions of MIRP, which ranked 0%, 25%, 50%, 75% and 100%, respectively.

**Figure 1:**
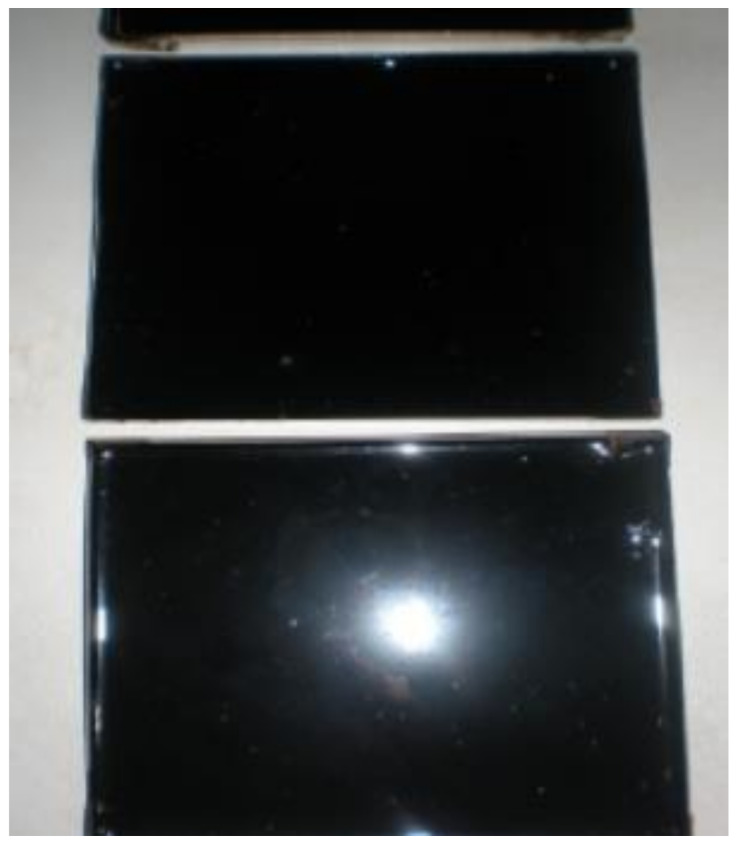
Asphalt slurry specimens.

The steps for making asphalt mortar are as follows: (i) Mix the limestone mineral powder and MIRP required by the pattern thoroughly and preheat them in a 180 °C oven. Dissolve the base asphalt which meets the quality requirement in a quantity of trichloroethylene. (ii) Blend the preheated MIRP and limestone mineral powder into the dissolved asphalt to make it evenly disperses in the base asphalt. (iii) Pour the mixed infrared powder asphalt into a 100 mm × 100 mm × 5 mm test mold placed on a flat table. (iv) Preliminary made specimens are placed in 80 °C oven for 6 h, ensuring the infrared powder asphalt specimens are under equal thickness film condition. (v) Place the baked specimens in the air to cool to room temperature. Figure 1 is the picture of asphalt slurry specimens.

**Figure 2:**
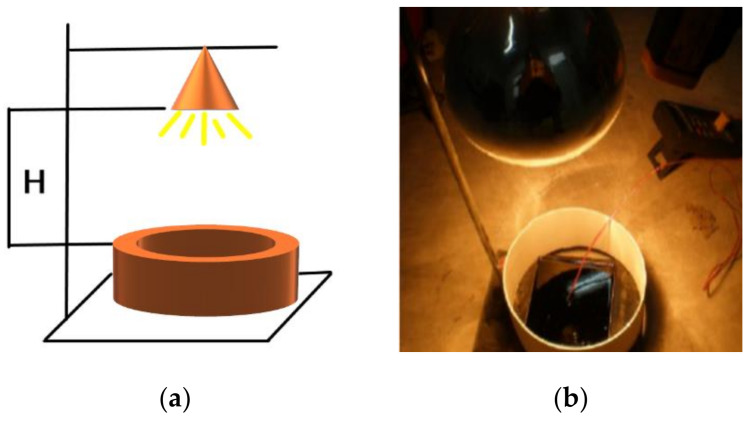
Asphalt slurry radiation testing device. (**a**) Schematic diagram; (**b**) Experimental installation

This study referred to the U.S. military standard MIL-E-46117A(MR), MIL-E-46136A(MR) as the basic principles and used the test system which can basically and accurately reflect the infrared powder asphalt-to-heat radiation performance [20,21]. Figure 2 is the asphalt slurry radiation testing device. An infrared heating lamp with a light source power of 250 watts was used to simulate solar radiation and the wavelength range of the infrared heating lamp was 700–2500 nm.

**Figure 3:**
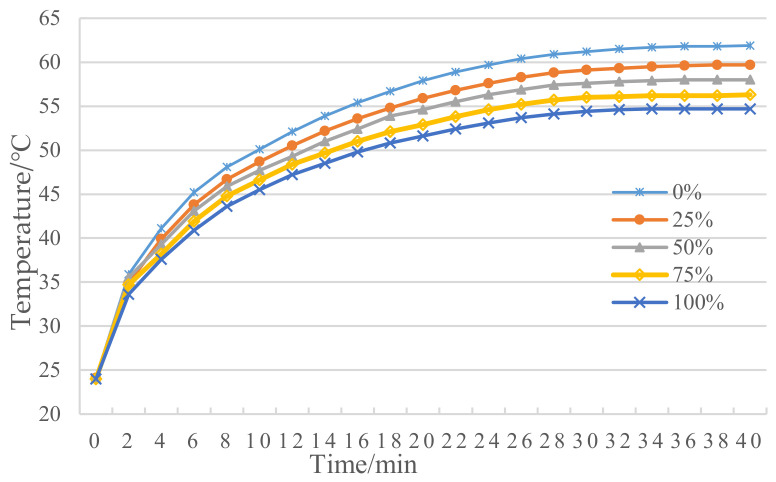
Asphalt slurry radiation test temperature rise curves.

In the asphalt slurry radiation test period, we controlled the infrared heating lamp’s distance H from the asphalt slurry surface for 20 cm, the sample surface and internal temperature were measured by infrared thermometer gun and numerical pyrometer, respectively, and the average was taken as the test data. The test was performed every two minutes until the temperature of the sample reached equilibrium. The asphalt slurry radiation test temperature rise curves of asphalt slurry with different proportion of MIRP are depicted in Figure 3.

As shown in Figure 3, it can be observed that the equilibrium temperature at the surface of the asphalt slurry decreases as the amount of MIRP doping increases. The equilibrium temperature of the non-modified-infrared-doped sample reached 61.9 °C, compared to 2.2 °C, 3.9 °C, 5.7 °C and 7.2 °C for the 25%, 50%, 75% and 100% modified-infrared-doped asphalt slurries, respectively. The equilibrium temperature of non-modified-infrared-doped asphalt mixture was reached in 40 min, while the time required to reach the equilibrium temperature was 38, 36, 34 and 34 min for asphalt pastes with 25%, 50%, 75% and 100% MIRP, respectively. It can be concluded that the incorporation of the MIRP is beneficial to the reduction of the equilibrium temperature of the asphalt slurry surface.

### 2.2. Infrared Thermal Radiation Test

In order to reveal the temperature characteristics of asphalt mortar mixed with different proportions of MIRP in SMA pavement, the MIRP was selected a special kind of limestone mineral powder, the total content of filler which consisted of MIRP and limestone mineral powder was maintained to a certain percent in this study (12%, Table 2). Therefore, the proportions of MIRP were changed to study its cooling characteristics under different MIRP dosing conditions. In the current study, the design of the proportion is based on SMA-13, with equal amount of MIRP replacing 0%, 25%, 50%, 75% and 100% of limestone mineral powder as filler, to make I–V type infrared thermal radiation asphalt mixture, respectively.

In this section, the SMA concrete (SMA-13) specimens were taken as the research object in this paper whose target MIRP content was set to be 0%, 3%, 6%, 9% and 12%, respectively, with 3 specimens per group. Thermal radiation test on Marshall specimens allows a comparison of the effect of MIRP on road materials from the difference between the surface temperature and the equilibrium temperature of the specimens. The aggregate gradation is shown in Table 5.

The infrared temperature measurement test device diagram is shown in Figure 2a. Test the vertical distance H from the infrared lamp to the surface of the test piece, adjust its size to 20 cm, turn on the infrared lamp, test the equilibrium temperature of the asphalt surface in 60 min. Considering that the extreme temperature of urban pavement can reach about 68 °C in summer, the height of H can be adjusted by the differential method to make it lower when the equilibrium temperature is lower than 68 °C; when the equilibrium temperature is higher than 68 °C, H is adjusted to make it higher. The test data are shown in Table 6. Therefore, it can be concluded that the infrared lamp height H is 23.5 cm when the surface temperature of the specimen reaches 68 °C for 60 min.

During the test, the infrared light was directed continuously at the surface of the specimen for 61 min and then switched off for 8 min. The initial temperature at the center of the upper and lower surface of the specimens was simultaneously measured. The infrared gun and numerical thermometer readings were taken every minute.

**Figure 4:**
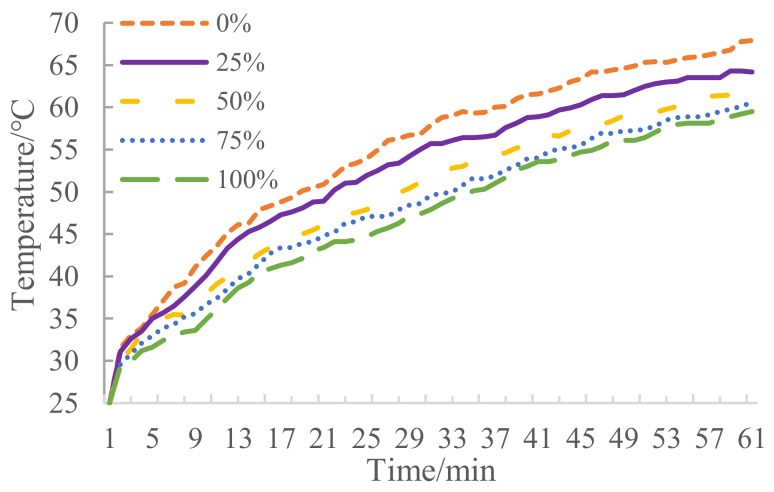
Surface warming curves.

**Figure 5:**
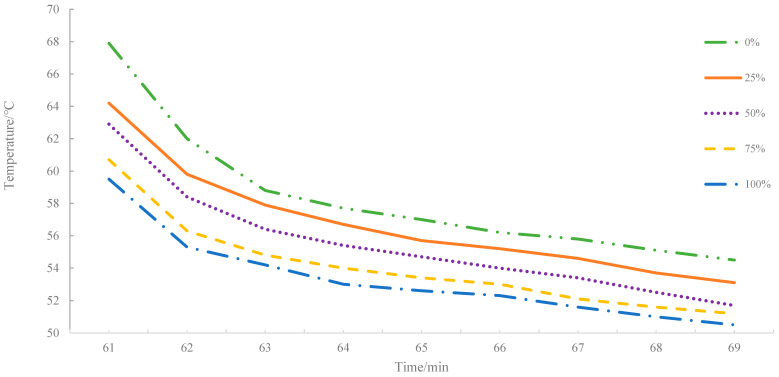
Surface cooling curves.

The surface warming curves and surface cooling curves of asphalt mixture with different proportion of MIRP are shown in Figure 4 and Figure 5, respectively. Based on Figure 4, with the increase of irradiation time, the surface temperature of the specimen is gradually increased and tends to temperature equilibrium. Additionally, as the MIRP content increases, the surface temperature of the mixture tends to increase more slowly; the higher the MIRP content, the lower the temperature increase.

Figure 5 indicates that with the increasing proportion of MIRP, the cooling time that is required for the surface temperature of the mixture to decrease to the same temperature difference diminishes. The main reason for the decrease of surface temperature is mainly due to the contact of air at a lower relative temperature without a radiation source. When the specimen surface decline tendency becomes moderate, with the increase amount of MIRP, the surface temperature is lower. Thus, it can be concluded that under the infrared radiation lamp, the specimens with MIRP can absorb less energy which accounts for low heat accumulation to a certain extent.

**Figure 6:**
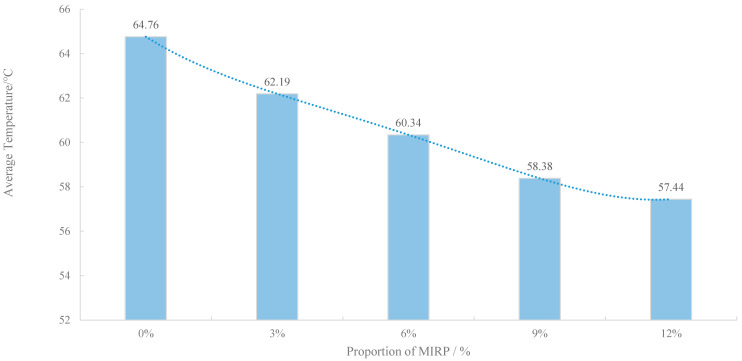
Equilibrium temperature of the specimens’ surface.

In order to facilitate the accuracy of the calculation, the test was taken at 52–61 min when the average temperature of the upper surface is the equilibrium temperature of the specimen, which is depicted in Figure 6. It indicates that the equilibrium temperature of the specimens decreased gradually with the increase of MIRP doping. The non-MIRP-doped specimens reached 66.59 °C, which is close to the extreme temperature of the actual summer pavement. When the MIRP was 3%, 6% and 9%, the equilibrium temperature of the specimen surface was 2.84 °C, 5.25 °C and 7.1 °C, respectively, which was lower than the non-infrared-powdered specimen. It suggests that the incorporation of MIRP can effectively reduce the surface temperature of the specimen and improve the high temperature stability of the mixture from the side. Meanwhile, the heat absorption of the mixture is reduced under the condition of equal irradiance, and the heat accumulation is reduced.

**Figure 7:**
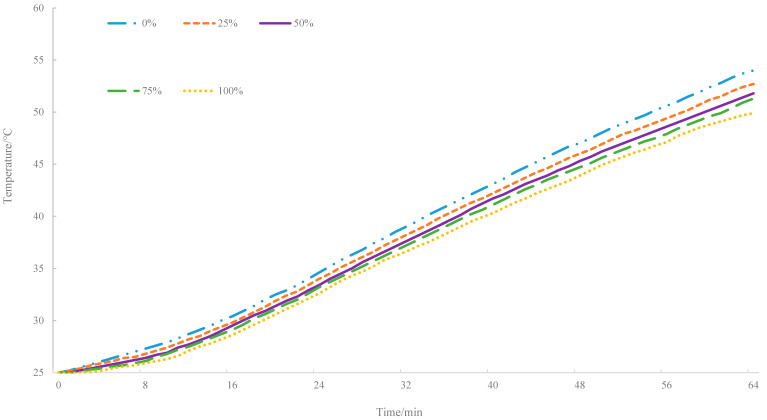
Bottom warming curves.

The bottom and side insulation devices were quipped in the infrared thermal radiation test, and the bottom heat absorption of the specimen had a hysteresis relative to the surface layer, leaving the specimens in a warming state even when the radiation source was turned off. The bottom warming curves of asphalt mixture with different proportion of MIRP are depicted in Figure 7. It clearly illustrates that the bottom temperature gradually decreases with increasing infrared powder doping according to Figure 7.

**Figure 8:**
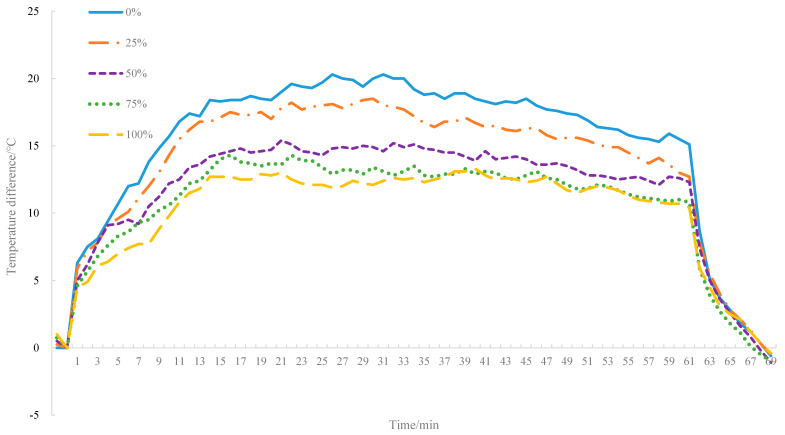
Residual curves.

Based on the analysis from Figure 7, the temperature rising of the bottom layer decreases with the increase of the proportions of MIRP in asphalt mixture, which, according to the heat transfer principle, may be related to two factors: the change in thermal conductivity and the difference in temperature gradient. Therefore, the difference between the surface and bottom temperature curves (referred to as residual curves) are plotted in Figure 8, and it can be found that throughout the test process, the temperature difference between the surface and bottom temperature of the asphalt specimens decrease with the proportion of MIRP increase.

### 2.3. X-ray Diffraction Test

**Figure 9:**
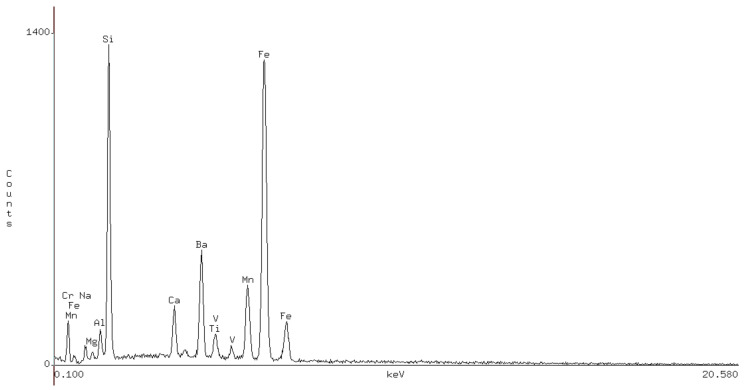
The infrared powder spectrum curve of modified infrared powder (MIRP).

The outward appearance of the MIRP made by this method is similar to ordinary black particulates and with a grain size of about 18–20 μm. X-ray diffraction tests were performed to study the chemical composition of the MIRP, and the test results are recorded in Table 7. The infrared powder spectrum curve of MIRP is shown in Figure 9.

The results in Table 7 and Figure 9 show that MIRP is mainly composed of nonmetallic oxides and metal oxides, of which the silicon oxide and iron oxide concentrations accounted for 35.83% and 34.64% of the total, respectively. In general, most experimented mixtures are about 30–60% silicon dioxide in the preparation of MIRP because the existence of silica making MIRP can decompose silicates at high temperature conditions and form a glass-phase structure that has improved infrared radiation performance gently. To clarify the performance of the MIRP, the infrared emissivity and wavelength were measured by an infrared radiation measuring instrument, and the results are shown in Table 8.

Here, F1 is the full wavelength integrated emission rate, F2 is the (8–14) μm integrated emission rate, F8 is the (14–25) μm integrated emission rate, and F3, F4, F5, F6 and F7 correspond to the center wavelengths of 8.45 μm, 9.50 μm, 10.60 μm, 12.00 μm and 13.50 μm, respectively. Those five models are narrow band emissions with a bandwidth of 1 μm. From the data in Table 1, the emissivity of MIRP in the 8–14 μm band can reach 0.94, while the emissivity at the full wavelength is only 0.92. The results also show that the emissivity outside the atmospheric window is lower than 0.92, therefore, the MIRP can be regarded as an alternative emission material.

Considering the different emission intensity of various materials in different wavelengths and maximizing the emission rate of MIRP in the whole waveband, it is essential to incorporate an appropriate amount of other oxides, such as magnesium oxide and alumina. Furthermore, the MIRP was treated with alkali to bond it to the acid anhydride and to enhance its activities in the asphalt mixture. In this experiment, Ca(OH)_2_ was used as the alkaline reagent, the ratio of reagent to water was 1:2, the MIRP was soaked in an alkali solution for about 3 h, and then dried to constant weight to yield the final alkali MIRP for further experiment.

### 2.4. Scanning Electron Microscopy Test

In order to further analyze the reasons for the improvement of the mixture performance after the MIRP replaced the limestone mineral powder, the microstructure analysis of the two fillers and the two asphalt mortars was carried out by SEM (Scanning Electron Microscopy).

**Figure 10:**
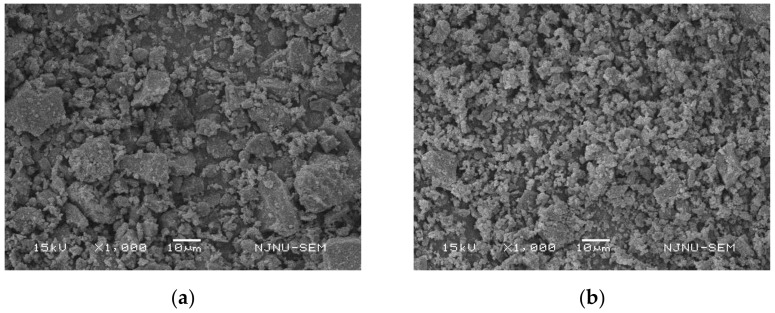
Microscopic photographs of limestone mineral powder and MIRP: (**a**) Limestone mineral powder; (**b**) MIRP.

In this paper, the scanning electron microscopy (SEM) was performed to detect the differences between MIRP and limestone mineral powder in asphalt mortar whose compound is shown in Table 4, and the test results are shown in Figure 10.

Figure 10 shows that the MIRP particles are smaller, more uniformly distributed, and have less aggregation than the limestone mineral powder. In this case study, the same weight of MIRP exhibited a greater specific surface area, which helped it to absorb asphalt and perform better.

**Figure 11:**
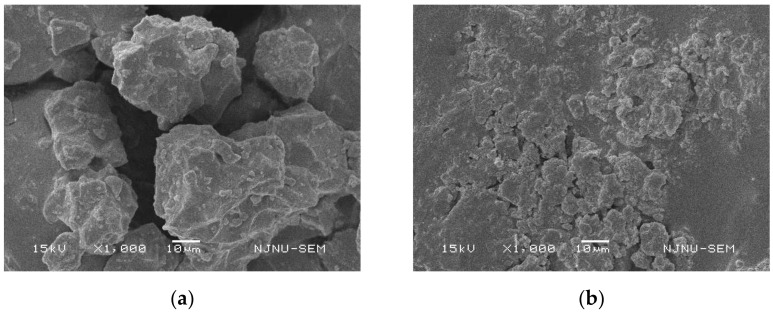
Microscopic photographs of asphalt mortar: (**a**) Mixed with limestone mineral powder; (**b**) Mixed with MIRP.

Similarly, SEM analyses were also conducted to detect the differences between asphalt mortar mixed with limestone mineral powder and asphalt mortar mixed with MIRP. The test results are depicted in Figure 11.

Figure 11 presents that the asphalt mortar with MIRP has a more compact structure and more uniform distribution than the limestone mineral powder asphalt mortar. In addition, the alkali-treated MIRP can absorb acidic asphalt and make it fully infiltrated into the voids and cracks, which can increase the contact area, make the asphalt mortar more cohesive, enhance the overall structural properties of the mixture, and improve the mechanical properties of the material to some extent.

### 2.5. Emissivity of Common Materials

Earlier studies reveal that the emissivity of most metallic and nonmetallic oxides can reach over 0.80 when the temperature reaches 100 °C, as shown in Table 9 [20].

Table 9 suggests that metal oxides, carbides, nitrides and borides are the kind of materials with high thermal radiation coefficients, and previous studies show that far-infrared emissivity of metallic and nonmetallic oxides is related to their purity and production processes [21]. Additionally, high-purity and high-quality materials can be obtained from screening tests, and the infrared emissivity at indoor temperature can be improved by using these materials and adding rare earth elements as activators [22].

## 3. Thermal Radiation Model

### 3.1. Establishment of the Thermal Radiation Model

The asphalt thermal equilibrium in the natural environment based on the radiant heat transfer theory can be expressed as follows [23]:

(2) $$q=q_{rs}+q_{ra}+q_{c}+q_{k}-q_{r}$$

Here, q is the net heat flux density of objects in the natural environment obtained, $q_{rs}$ and $q_{ra}$ are the absorbed solar radiation and atmospheric radiation heat flux, $q_{c}$ and $q_{k}$ are the net heat flux and thermal convection obtained, respectively. $q_{r}$ is the radiation emitted by the surface heat flux.

To achieve the equilibrium state of asphalt surface temperature, make q = 0. Since the temperature distribution of the atmosphere is not uniform, and the absorption and emission of the atmosphere is selective, for longwave radiation, there exists an atmospheric window only between 8–14 μm, and the temperature of the cosmic space outside the atmosphere is very low, about 3 K. Therefore, when calculating the radiation heat exchange between the ground object and the sky, the atmospheric radiation and the sky radiation are considered as black bodies of some effective sky temperature [24] where the effective sky temperature is lower than the ground atmospheric temperature $T_{s}$ for simplification.

Considering that the temperature difference between asphalt pavement and near-ground air is very small [25,26], the thermal convection obtained can be ignored, making $q_{k}$ = 0 to obtain the following asphalt pavement heat balance equation:

(3) $$q_{rs}+q_{ra}+q_{c}-q_{r}=0$$

**Figure 12:**
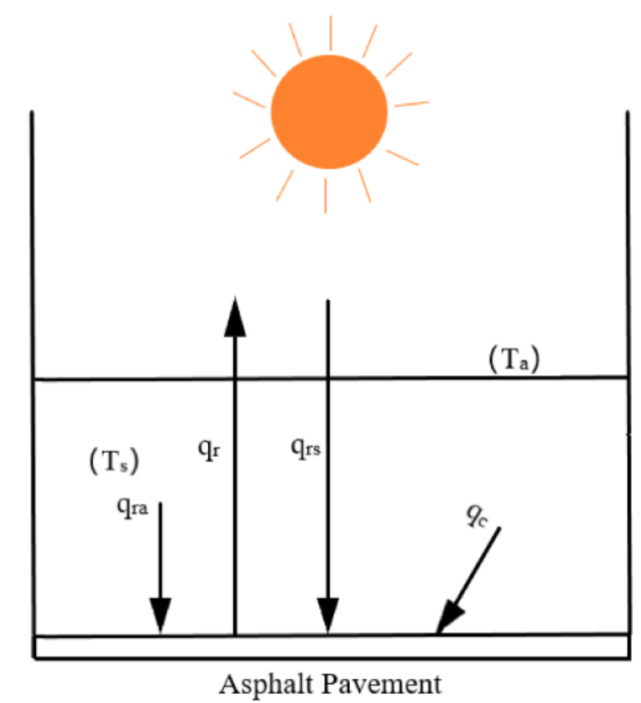
The pavement cooling model.

The pavement cooling model is shown in Figure 12.

Assuming that when the solar radiation penetrates the atmosphere and irradiates the asphalt pavement surface, the pavement equilibrium temperature is $T_{e}$, the atmospheric temperature is T, the sky effective temperature is $T_{s}$, the solar irradiance is $G_{s}$, the convective heat transfer coefficient between the pavement and the near-surface atmosphere is h, the shortwave absorptivity of the asphalt pavement is $a_{s}$, and the longwave emissivity is ε, then the following formula can be obtained based on the radiant heat transfer theory:

(4) $$q_{rs}=G_{s}a_{s}$$

(5) $$q_{rs}=\varepsilon_{1}\sigma T_{s}{}^{4}$$

(6) $$q_{c}=h\left(T-T_{e}\right)$$

(7) $$q_{rs}=\varepsilon_{1}\sigma T_{e}{}^{4}$$

Taking the Stefan–Boltzmann constant as $\sigma =5.73\times 10^{-8}W/\left(m^{2}\cdot K\right)$, the equilibrium temperature model of asphalt pavement can be obtained as follows:

(8) $$5.73\times 10^{-8}\varepsilon_{1}\left(T_{e}{}^{4}-T_{s}{}^{4}\right)=G_{s}a_{s}+h\left(T-T_{e}\right)$$

### 3.2. Boundary Conditions of the Thermal Radiation Model

(1) Sky effective temperature $T_{s}$.

Effective atmospheric temperature is mainly affected by the infrared radiation transmissivity in the atmospheric window (*x*) and atmospheric temperature (*T*) [27]. However, in general, because the ground longwave radiation only exists in the atmospheric window of 8 μm–14 μm, which only accounts for a very small part of the ground longwave radiation, when calculating the effective sky temperature, the influence of this part is rarely considered, under the same conditions of permeability, with temperature changes, the empirical formula:

(9) $$T_{s}=0.052T^{1.5}$$

From the blackbody radiation function [27], the radiation energy between a given waveband $\lambda_{1}\sim \lambda_{2}$ can be expressed as follows:

(10) $$F_{\lambda_{2}-\lambda_{1}}=F_{0-\lambda_{2}T}-F_{0-\lambda_{1}T}$$

(11) $$F_{0-\lambda_{1}T}=\overset{\lambda T}{\underset{0}{\int }}\frac{e_{\lambda b}\left(\lambda ,T\right)}{\sigma T^{5}}d\left(\lambda T\right)$$

(12) $$\frac{e_{\lambda b}\left(\lambda ,T\right)}{\sigma T^{5}}=\frac{2\pi c_{1}}{{\left(\lambda T\right)}^{5}\left(e^{c_{2}/\left(\lambda T\right)}-1\right)}$$

**Figure 13:**
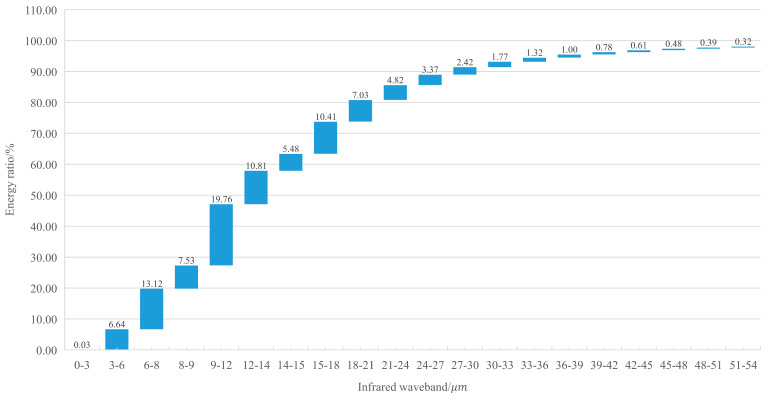
Distribution of radiant energy in different infrared waveband.

It can be seen from Formula (12) that $F_{0-\lambda_{1}T}$ is only a function of λT, and the road surface temperature in the high temperature season is 60 degrees Celsius, which is 333 K. The blackbody radiation function table was checked to compile the radiation energy distribution table of different bands, as shown in Figure 13.

The proportion of radiation in the long wave band (λ∈(3 μm~∞)):

$$\begin{array}{c} F_{3-\infty }=1-F_{0-3}=99.968\%\\ \%F_{3-66}=F_{3-\infty }-\left(1-F_{0-66}\right)=99.968\%-\left(1-98.885\%\right)=98.853\%\\ F_{8-14}=F_{0-14}-F_{0-8}=58.891\%-19.789\%=38.102\% \end{array}$$

The concept of band radiant energy distribution rate (%/μm) was introduced:

(13) $$v_{\lambda_{1}-\lambda_{2}}=F_{\lambda_{1}-\lambda_{2}}/\left(\lambda_{1}-\lambda_{2}\right)$$

$$v_{8-14}=6.35\%/\mu m$$

$$v_{3-66}=1.569\%/\mu m$$

Figure 13 presents that when the pavement temperature reaches 333 K, the radiation energy of ordinary asphalt pavement in the infrared window accounts for 38.102% of the total radiation energy.

As the low thermal accumulation asphalt mixture is mixed with infrared powder, it will certainly improve the overall transmission performance of the mixture in the infrared window band. Furthermore, its transmittance in the atmospheric window band reaches 94%, and since the IR emissivity varies with temperature, its transmittance is *x* (%), assuming its atmospheric temperature remains constant:

(14) $$x\sigma {T_{\varepsilon }}^{4}+\left(1-x\right)({\sigma T}_{\varepsilon }{}^{4}-{\sigma T}^{4})=\sigma \left(T_{\varepsilon }{}^{4}-T_{S}{}^{4}\right)$$

(15) $$\left(1-x\right)T^{4}=T_{s}{}^{4}$$

**Figure 14:**
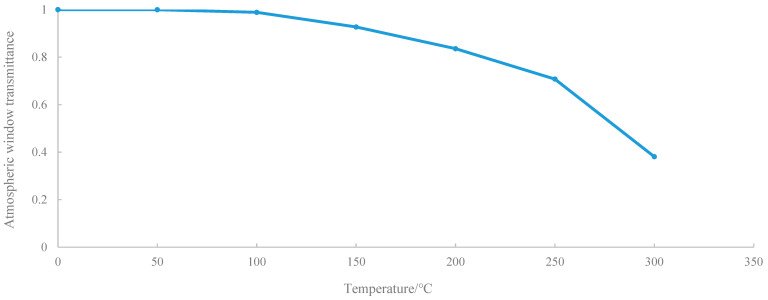
Atmospheric window transmittance versus effective sky temperature curve.

According to the empirical Formula (8), the relationship curve between the fitted atmospheric window permeability and effective sky temperature is shown in Figure 14, and the sky effective temperature can be expressed as follows:

(16) $$T_{S}=0.552\times {\left(\frac{1-x}{1-0.381}\right)}^{\frac{1}{4}}T^{\frac{3}{2}}$$

(2) Solar irradiance $G_{s}$.

When the temperature is 5762 K (5488.85 °C), the solar radiation spectrum is equivalent to the blackbody emission [28], the solar irradiance at this time is also called the solar constant, and its value is 1353 $W/m^{2}$. When solar radiation passes through the atmosphere, it is scattered by various molecules and dust in the air and becomes absorbed by ozone, water vapor and carbon dioxide at a certain band of energy. Thus, when the radiation reaches the ground, the solar irradiance on the earth’s surface usually decreases to less than 1000 $W/m^{2}$. Statistical data show that Nanjing is close to the 32nd parallel of north latitude, and the solar irradiance is 610.2 $W/m^{2}$ [29,30].

(3) Atmospheric temperature *T.*

Near-surface atmospheric temperature mainly depends on the magnitude of longwave radiation energy, and declines as the altitude increases. Generally, near-surface atmospheric temperature refers to air temperature between 1.25 and 2 m above the ground. Statistical data [31] showed that the average summer extreme maximum temperature in Nanjing is 310 K (36.85 °C).

(4) Convective heat transfer coefficient h.

Convective heat transfer coefficient refers to the process of heat exchange between pavement and atmosphere, because of the existence of temperature variations. The empirical formula proposed by the American Society of Heating, Refrigeration and Air Conditioning Engineers (ASHRAE) [32] is as follows:

(17) $$h=av^{2}+bv+c$$

In Formula (13), *v* represents wind speed, and *a*, *b*, and *c* are the empirical constants related to the type of pavement. For asphalt pavement, *a* = 0, *b* = 1.874, and *c* = 10.788 when *v* = 0 *m/s*, *h* = 10.788 $W/\left(m^{2}\cdot K\right)$.

(5) Asphalt pavement shortwave absorptivity *a_s_*_._

Generally, asphalt pavement has a strong absorption rate of shortwave radiation, and its absorptivity mainly depends on the blackness of pavement materials. As the blackness increase, the amount of radiation absorbed by the pavement increases. Due to the deep color, the asphalt pavement can absorb most of the radiation. Previous studies have shown that the shortwave absorptivity of ordinary asphalt pavement is about 0.8–0.85 [33] and the shortwave absorptivity of new dust-free asphalt concrete pavement is close to 0.93 [34].

(6) Asphalt pavement longwave emissivity $\varepsilon_{1}$.

The emissivity of an object is the ratio of the object’s emissivity to that of a blackbody of the same temperature. The surface emissivity is a property of the surface of the object itself and depends on the surface temperature, emission direction and emission wavelength. For asphalt pavement, because of its rough surface, equivalent to a diffuse gray surface, it was found that its normal full emissivity can reach about 0.9 [35]. It can also be found from the earth’s radiation spectrum that the longwave radiation energy on the ground is mainly distributed in the infrared band of 5–25 μm, with the highest radiation intensity around 10 μm. Due to the incorporation of MIRP, and its radiation wavelength being 8–13.5 μm, which is located near 10 μm, the radiation performance of the asphalt mixture will be further improved.

## 4. Thermal Radiation Model Analysis

### 4.1. Influence of Infrared Radiation Transmissivity

From the energy balance Equation (8) of asphalt pavement,

(18) $$5.73\times 10^{-8}\varepsilon_{1}(T_{e}^{4}-T_{s}{}^{4})=G_{s}a_{s}+h(T-T_{e})$$

(19) $$5.157\times 10^{-8}T_{e}^{4}+10.788T_{e}+686.39x-4579.85=0$$

Considering *T* = 310K, *G_s_* = 610.2 $W/m^{2}$, *h* = 10.788 $W/\left(m^{2}\cdot K\right)$ *a_s_* = 0.9, $\varepsilon_{1}=0.9$, the equilibrium temperature equation of asphalt pavement can be simplified as follows:

(20) $$T_{e}{}^{4}+2.0919\times 10^{8}T_{e}+\left(1.331x-883.1\right)\times 10^{8}$$

**Figure 15:**
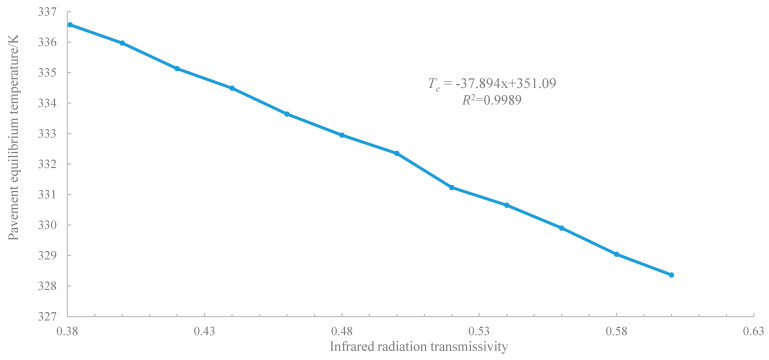
Fitting curves between pavement equilibrium temperature and infrared radiation transmissivity.

By calculating the equilibrium temperature under different transmittance conditions, the fitting curves between the pavement equilibrium temperature and infrared radiation transmissivity of asphalt pavement are obtained as shown in Figure 15.

Figure 15 presents that the relationship between pavement temperature and infrared radiation transmissivity is linear, and the formula of the relationship curve is as follows:

(21) $$T_{e}=-37.894x+351.09$$

Thus, a 1% increase in the infrared radiation transmissivity results in a 0.379 °C decrease in pavement equilibrium temperature, indicating that improving infrared radiation emissivity does have an effect on reducing pavement temperature.

### 4.2. Influence of Shortwave Absorptivity and Longwave Emissivity

The analysis of boundary conditions shows that when the wind speed is 0 *m/s* and the atmospheric pressure is the standard atmospheric pressure, considering *T* = 310 K (36.85 °C), *G_s_* = 610.2 $W/m^{2}$, *h* = 10.788 $W/(M^{2}\cdot K)$, then the equilibrium temperature equation of asphalt pavement can be simplified as follows:

(22) $$T_{e}{}^{4}+1.8827\times 10^{8}\varepsilon_{1}{}^{-1}T_{e}-\left(106.5a_{S}+583.64+82.4\varepsilon_{1}\right)\times 10^{8}\varepsilon_{1}{}^{-1}=0$$

Considering the pavement equilibrium temperature affected by several common shortwave absorptivity and longwave radiation emissivity, the calculation results are shown in Table 10.

**Figure 16:**
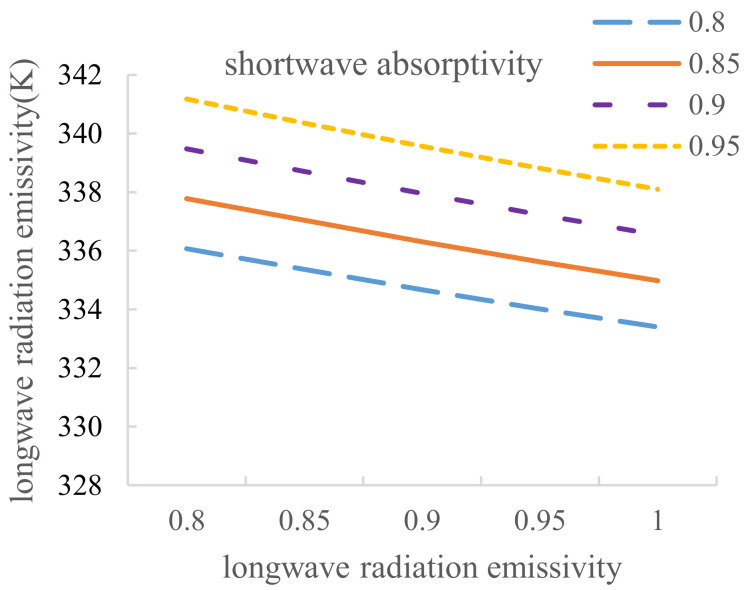
Relationship between pavement equilibrium temperature and longwave radiation emissivity.

**Figure 17:**
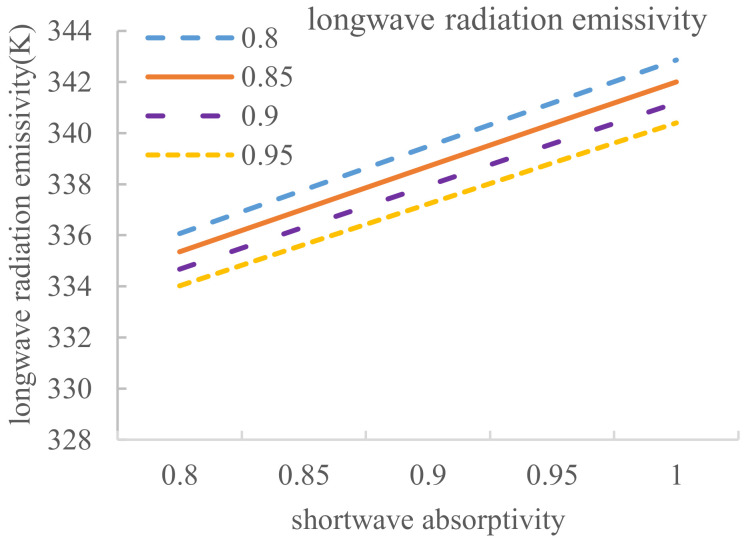
Relationship between pavement equilibrium temperature and the shortwave absorptivity.

Table 10 reveals that the relationship between pavement equilibrium temperature and longwave radiation emissivity and the relationship between pavement equilibrium temperature and the shortwave absorptivity can be obtained, and these results are depicted in Figure 16 and Figure 17.

Figure 16 shows that when the shortwave absorptions are 0.8, 0.85, 0.9 and 0.95, the corresponding curve slopes are −15.4, −14.7, −14.02 and −13.36, respectively, which are all lower than 0. Thus, the equilibrium temperature declines with increasing longwave radiation emissivity. In addition, because the four curves are roughly parallel, the relationship between pavement equilibrium temperature and longwave radiation emissivity can be regarded as a linear relationship, and the average slope of −14.37 can be taken as a comprehensive slope. In other words, a 1% increase in the longwave radiation emissivity results in a 0.1437 °C decrease in pavement equilibrium temperature.

Similarly, Figure 17 shows that when the longwave emissivity values are 0.8, 0.85, 0.9, 0.95 and 1, the corresponding curve slopes are 34.06, 33.32, 32.68, 32 and 31.32, respectively, which are all greater than 0. Thus, equilibrium temperature increases with increasing shortwave absorptivity. In addition, because the five curves are roughly parallel, the relationship between pavement equilibrium temperature and shortwave absorptivity can be regarded as a linear relationship, and the average slope 32.68 can be taken as a comprehensive slope. In other words, a 1% decrease in the shortwave absorptivity results in a 0.3268 °C decrease in pavement equilibrium temperature.

### 4.3. Cooling Performance Analysis

Based on the former results and analysis, the cooling effect of the asphalt mixture with different proportions of MIRP can be summarized in the following analysis.

According to the results of infrared radiation tests in Section 2.1, it can be concluded that the incorporation of the MIRP is beneficial to the reduction of the equilibrium temperature of the asphalt slurry surface. The surface warming curves indicate that the surface temperature of the specimens gradually increased and tend to reach the temperature equilibrium with the increase of irradiation time. Meanwhile, the surface cooling curves suggest that as the MIRP content increases, the surface temperature of the mixture tends to increase more slowly, and the higher the MIRP content, the lower the temperature increase. Additionally, it can be concluded that as the MIRP proportion increases, the cooling time required for the surface temperature decrease as the same temperature difference is diminished. These results demonstrate that the asphalt mixtures added to MIRP can absorb less energy which accounts for low heat accumulation.

The thermal radiation model indicated that the pavement equilibrium temperature was mainly influenced by infrared radiation transmissivity, shortwave absorptivity and longwave emissivity, and that adding MIRP to the asphalt mixture can improve the performance of asphalt pavement to a certain extent.

The pavement surface equilibrium temperature combined with the MIRP in asphalt mixture decreases with the increase of the longwave emissivity, and it diminishes with the decrease of the shortwave absorptivity. Additionally, the emissivity of MIRP in the infrared band can reach 92%, while that of ordinary asphalt pavement is only about 80%. Thus, the infrared radiation transmissivity and the longwave emissivity of the mixture can be improved by adding MIRP. In addition, previous tests have shown that shortwave absorptivity is mainly related to the surface depth of color and roughness of the mixture, and a deeper color and higher roughness usually means greater shortwave absorptivity. Microscopic experiments prove that the addition of MIRP has little effect on the color of the mixture, but has a significant effect on the roughness of the mixture. The asphalt mixture with MIRP has smaller holes and cracks, a smoother surface, and lower roughness than the ordinary asphalt mixture. Therefore, the application of MIRP in asphalt pavement can reduce the pavement equilibrium temperature by reducing the shortwave absorptivity of the pavement to a certain degree.

In general, the application of MIRP in asphalt pavement can reduce the pavement temperature to some extent, which provides an effective way to alleviate the urban heat island effect. In further study, different kinds of the modified infrared powder will be considered to study the cooling mechanism in asphalt mixture.

## 5. Conclusions

Based on the asphalt slurry cooling performance test and infrared thermal radiation test, the cooling performance of MIRP was obtained in this research. X-ray diffraction analysis and scanning electron microscopy test (SEM) were conducted to analyze the chemical composition and the microstructure of MIRP, respectively. According to the radiant heat transfer theory, a thermal radiation model of the pavement equilibrium temperature was established by microscopic and chemical analysis to study the influence of thermal radiation asphalt mixture and reveal its cooling performance at last. The main conclusions of this paper can be drawn as follows:

1. X-ray diffraction tests show that the main components of MIRP are metal oxides and nonmetallic oxides, in which the content of silicon oxide and iron oxide are 35.32% and 34.64%, respectively, and the proportion is much higher than that of other components. The existence of these materials helps improve infrared emissivity of the asphalt pavement.
2. The SEM test shows that compared with the limestone mineral powder, the MIRP has smaller particles and less agglomeration. Compared with limestone mineral powder asphalt mortar, the asphalt mortar with MIRP has a more compact structure and more uniform distribution, enhancing the overall structural performance of the mixture.
3. The addition of MIRP can improve the infrared radiation transmissivity of asphalt pavement, and the asphalt pavement equilibrium temperature decreased with increased infrared radiation transmissivity of the asphalt mixture. A 1% increase in infrared radiation transmissivity results in a 0.379 °C decrease in asphalt pavement equilibrium temperature.
4. The addition of MIRP can improve the longwave emissivity of asphalt pavement, and the equilibrium temperature of asphalt pavement decreases as the percentage of longwave emissivity of the pavement increases. A 1% increase in the longwave emissivity results in a 0.1437 °C decrease in asphalt pavement equilibrium temperature.
5. The addition of MIRP can decrease the shortwave absorptivity of asphalt pavement, and the asphalt pavement equilibrium temperature decreased with decreased shortwave absorptivity of the pavement. A 1% decrease in the shortwave absorptivity results in a 0.3268 °C decrease in asphalt pavement equilibrium temperature.

## Figures and Tables

**Table 1:** The surface dry relative density of coarse aggregates.

Sieve Size (mm)	Quality in Water (g)	Dry Quality (g)	Drying Quality (g)	Apparent Relative Density (g/cm^3^)	Surface Dry Relative Density (g/cm^3^)	Relative Density of Bulk Volume (g/cm^3^)
4.75	633.2	1002.1	999.6	2.728166	2.716454	2.709677
9.5	633.7	1001.8	999.4	2.732841	2.721543	2.715023
13.2–16	633	1001.1	999.8	2.725736	2.719641	2.71611

**Table 2:** The apparent relative density of fine aggregates.

Sieve Size (mm)	Fine Aggregates Quality (g)	Cylinder Quality (g)	Cylinder + Water (g)	Cylinder + Water + Fine Aggregates (g)	Apparent Relative Density (g/cm^3^)
4.75–2.36	238	365.8	861.7	1011.2	2.689266
2.36–1.18	300	471.1	1454.4	1642.1	2.671416
1.18–0.6	300	471	1455.5	1642.9	2.664298
0.6–0.3	200	471.1	1456.7	1581.6	2.663116
0.3–0.15	100	103.3	449.5	511.9	2.659574
0.15–0.075	100	103.3	449.8	512.1	2.65252
limestone mineral powder	160	103.3	450.1	549.5	2.640264
MIRP	100	103.4	450.1	519.8	3.30033

**Table 3:** The infrared emissivity and wavelength.

Sample Name	Normal Specific Emissivity Value							
MIRP	F_1_	F_2_	F_3_	F_4_	F_5_	F_6_	F_7_	F_8_
	0.92	0.94	0.93	0.94	0.94	0.95	0.94	0.93

**Table 4:** Asphalt slurry specimen proportions.

Sample Proportions	0%	25%	50%	75%	100%
asphalt (g)	20	20	20	20	20
limestone mineral powder (g)	40	30	20	10	0
MIRP (g)	0	10	20	30	40

**Table 5:** Aggregate gradation (passing percent (%)).

Sieve Size (mm)	16	13.2	9.5	4.75	2.36	1.18	0.6	0.3	0.15	Mineral Powder	Infrared Powder
Type I	100	95.0	63.0	27.0	21.0	19.0	16.0	14.0	13.0	12	0
Type II	100	95.0	63.0	27.0	21.0	19.0	16.0	14.0	13.0	9	3
Type III	100	95.0	63.0	27.0	21.0	19.0	16.0	14.0	13.0	6	6
Type IV	100	95.0	63.0	27.0	21.0	19.0	16.0	14.0	13.0	3	9
Type V	100	95.0	63.0	27.0	21.0	19.0	16.0	14.0	13.0	0	12

**Table 6:** Infrared lamp height and specimen surface temperature.

H (cm)	20	22	25	30	23.5
T (°C)	74	71	64	52	68

**Table 7:** Composition and proportion of MIRP.

Type of Material	SiO_2_	Fe_2_O_3_	TiO_2_	MnO	Al_2_O_3_	Na_2_O	CaO	MgO	V_2_O_5_	Cr_2_O_3_	Others
Mass ratio (%)	35.83	34.64	8.51	7.43	3.35	2.89	2.71	1.85	0.91	0.82	0.64

**Table 8:** Infrared emissivity of MIRP at different wavelengths.

Wavelengths	F1	F2	F3	F4	F5	F6	F7	F8
Infrared emission rate	0.92	0.94	0.93	0.94	0.94	0.95	0.94	0.93

**Table 9:** Emission rates of common metal oxide and non-metal oxide (5–25 μm wideband) when temperature is higher than 100 °C.

Type of Material	Emissivity	Type of Material	Emissivity	Type of Material	Emissivity
Al_2_O_3_	0.88	Cr_2_O_3_	0.79	ZnO	0.83
CeO_2_	0.79	Co_2_O_3_	0.81	SiO_2_	0.83
Fe_2_O_3_	0.74	MgO	0.8	Mullite	0.82
Sb_2_O_3_	0.87	SiC	0.81	Sericite	0.8
ZrO_2_	0.82	TiO_2_	0.82	Kaolin	0.79

**Table 10:** Equilibrium temperature affected by common shortwave absorptivity and longwave radiation emissivity.

*T_e_*/K	𝛼_s_ = 0.8	𝛼_s_ = 0.85	𝛼_s_ = 0.9	𝛼_s_ = 0.95
*ε*_1_ = 0.8	336.07	337.78	339.48	341.18
*ε*_1_ = 0.85	335.36	337.04	338.70	340.36
*ε*_1_ = 0.9	334.67	336.31	337.95	339.57
*ε*_1_ = 0.95	334.02	335.63	337.23	338.82
*ε*_1_ = 1.0	333.40	334.98	336.54	338.10

## Data Availability

All data is included in the article, no additional data can be provided.
